# Heart Rate Variability and Global Longitudinal Strain for Prognostic Evaluation and Recovery Assessment in Conservatively Managed Post-Myocardial Infarction Patients

**DOI:** 10.3390/jcm13185435

**Published:** 2024-09-13

**Authors:** Carina Bogdan, Adrian Apostol, Viviana Mihaela Ivan, Oana Elena Sandu, Ion Petre, Oana Suciu, Luciana-Elena Marc, Felix-Mihai Maralescu, Daniel Florin Lighezan

**Affiliations:** 1 Department VII, Internal Medicine II, Discipline of Cardiology, “Victor Babeş” University of Medicine and Pharmacy, Eftimie Murgu Sq. No. 2, 300041 Timişoara, Romania; carina.bogdan@umft.ro (C.B.); ivan.viviana@umft.ro (V.M.I.); oana.ciolpan@umft.ro (O.E.S.); 2 Centre for Molecular Research in Nephrology and Vascular Disease, Faculty of Medicine “Victor Babeş”, 300041 Timișoara, Romania; marc.luciana@umft.ro (L.-E.M.); mihai.maralescu@umft.ro (F.-M.M.); 3 Department of Functional Sciences, Medical Informatics and Biostatistics Discipline, “Victor Babeş” University of Medicine and Pharmacy, Eftimie Murgu Sq. No. 2, 300041 Timişoara, Romania; petre.ion@umft.ro; 4 Department of Microbiology, “Victor Babes” University of Medicine and Pharmacy, 300041 Timișoara, Romania; suciu.oana@umft.ro; 5 Department of Internal Medicine II, Discipline of Nephrology, “Victor Babeș” University of Medicine and Pharmacy, 300041 Timișoara, Romania; 6 Department V, Internal Medicine I, Discipline of Medical Semiology I, “Victor Babeș” University of Medicine and Pharmacy, 300041 Timișoara, Romania; dlighezan@umft.ro; 7 Center of Advanced Research in Cardiology and Hemostaseology, “Victor Babeş” University of Medicine and Pharmacy, Eftimie Murgu Sq. No. 2, 300041 Timișoara, Romania

**Keywords:** heart rate variability, autonomic dysfunction, global longitudinal strain, myocardial infarction

## Abstract

**Background**: Heart rate variability (HRV) is the fluctuation in the time intervals between adjacent heartbeats. HRV is a measure of neurocardiac function that is produced by dynamic autonomic nervous system (ANS) processes and is a simple measure that estimates cardiac autonomic modulation. **Methods**: The study included 108 patients admitted to the Coronary Intensive Care Unit with acute myocardial infarction (AMI) who did not undergo primary percutaneous transluminal coronary angioplasty (PTCA) or systemic thrombolysis and followed conservative management. All patients underwent detailed clinical, biological, and paraclinical assessments, including evaluation of HRV parameters and echocardiographic measurements. The analysis of RR variability in both time and frequency domains indicates that the negative prognosis of patients with AMI is associated with an overall imbalance in the neuro-vegetative system. The HRV parameters were acquired using continuous 24 h electrocardiogram (ECG) monitoring at a baseline, after 1 month, and 6 months. **Results**: Our analysis reveals correlations between alterations in HRV parameters and the increased risk of adverse events and mortality after AMI. The study found a significant improvement in HRV parameters over time, indicating better autonomic regulation post-AMI. The standard deviation of all RR intervals (SDNN) increased significantly from baseline (median 75.3 ms, IQR 48.2–100) to 1 month (median 87 ms, IQR 55.7–111) and further to 6 months (median 94.2 ms, IQR 67.6–118) (*p* < 0.001 for both comparisons). The root mean square of successive difference of RR (RMSSD) also showed significant increases at each time point, from baseline (median 27 ms, IQR 22–33) to 1 month (median 30.5 ms, IQR 27–38) and from 1 month to 6 months (median 35 ms, IQR 30–42) (*p* < 0.001 for all comparisons), indicating enhanced parasympathetic activity. Moreover, changes in HRV parameters have been associated with impaired left ventricle ejection fraction (LVEF) and global longitudinal strain (GLS), indicating a relationship between autonomic dysfunction and myocardial deformation. GLS values improved from a baseline median of −11% (IQR 5%) to −13% (IQR 4%) at 6 months (*p* < 0.001), reflecting better myocardial function. **Conclusions**: HRV parameters and cardiac performance analysis, especially using GLS, offer a solid framework for evaluating recovery and predicting adverse outcomes post-MI.

## 1. Introduction

The sympathetic nervous system (SNS) and the parasympathetic nervous system (PNS) are the two primary components of the autonomic nervous system (ANS) that work together to mediate the body’s hemodynamic adjustments. Heart rate variability (HRV) is a noninvasive method for assessing cardiac autonomic modulation [1]. In patients who have experienced a myocardial infarction (MI) there is typically increased sympathetic activity and/or decreased parasympathetic activity, leading to reduced HRV. This reduction in HRV is associated with an increased risk of adverse events and mortality [2,3].

Based on 2019 data from the European Society of Cardiology, an estimated 113 million people worldwide were living with cardiovascular diseases. In Romania, according to general practitioners, 1 in 4 individuals, which amounts to over 4 million people, were reported to have cardiovascular disease [4].

The intrinsic firing rate of the sinoatrial node is modulated by the actions of the PNS through the vagus nerve, and the SNS through the sympathetic nerves. Consequently, the heart rate at any given time reflects the balance between these two divisions of the ANS [5]. HRV assesses the differences in the variability of the time intervals between consecutive heartbeats [2,5]. HRV serves as a sensitive indicator of overall system complexity and adaptability [6]. Time domain measures provide an indication of the overall extent of HRV. Extremely low values are indicative of true autonomic dysfunction, whereas elevated values may either reflect healthy autonomic function or, conversely, a highly erratic heart rhythm. Similarly, frequency domain measures display comparable characteristics. Low values suggest diminished autonomic regulation [7,8].

Multiple studies conducted over the last few decades have consistently shown that reduced HRV, indicative of cardiac ANS dysfunction, is closely linked to an increased likelihood of severe ventricular arrhythmias and mortality among patients who have experienced an acute myocardial infarction (AMI). The importance of HRV as a prognostic tool was initially recognized when research found that individuals with lower HRV following AMI had significantly higher rates of in-hospital mortality. Certain time-domain HRV metrics, such as the standard deviation of all normal RR intervals (SDNN), have been identified as independent predictors of long-term survival after an AMI [6,7]. Further studies have indicated that diminished HRV is not just a general marker of cardiac risk, but also a specific predictor of sudden cardiac death (SCD). The prognostic relevance of HRV also appears to be influenced by the timing of its measurement following an AMI, with early assessments (within the first two weeks) demonstrating a strong correlation with mortality risk. This variability over time highlights the evolving nature of autonomic function recovery post-MI, implying that repeated HRV assessments could provide critical insights into ongoing patient risk. Given the dynamic changes in the arrhythmic substrate after AMI, it is recommended to conduct HRV measurements early and then at subsequent intervals to better evaluate the risk of life-threatening arrhythmias [6,7,9,10,11]. However, there is limited understanding of how HRV parameters change over time in patients who have not undergone primary percutaneous coronary intervention (PCI) or thrombolysis, especially in those managed conservatively.

Heart dysautonomia, which is defined by the ANS’s inability to maintain cardiovascular homeostasis, can cause a variety of symptoms and repercussions that impair cardiovascular health and increase the risk of cardiovascular disease. HRV is frequently utilized to evaluate the impact of autonomic dysfunction on a range of conditions and as a prognostic indicator for heart disorders. Reduced HRV has been shown to independently predict higher mortality rates and serve as a risk factor for sudden death, indicating autonomic dysregulation [5,12,13]. Yet, the specific temporal dynamics of HRV recovery and its correlation with cardiac remodeling in the conservatively managed post-MI population remain underexplored.

Left ventricular (LV) function is a strong predictor of long-term survival in patients with cardiac diseases. Left ventricular ejection fraction (LVEF) is the most commonly used echocardiographic parameter to quantify LV systolic function. Speckle-tracking imaging is a relatively new technique for evaluating LV function with high temporal and spatial resolution by measuring myocardial strain. Global longitudinal strain (GLS) quantifies the degree of myocardial deformation and has become an important tool for assessing LV function in clinical settings. Compared with conventional methods, GLS is more sensitive and accurate in detecting subclinical cardiac changes [14,15,16]. LV GLS is a robust and sensitive measure of myocardial function and fibrosis, with significant prognostic value for various cardiac pathologies. GLS offers a more precise evaluation of myocardial contractile function, enabling the detection of early and subtle impairments, when compared with LVEF. This makes GLS particularly valuable for detecting early-stage cardiac dysfunction [17]. GLS offers independent and additional prognostic insight into the long-term risk of cardiovascular morbidity and mortality evaluation [18,19]. In patients post-AMI, GLS allows for the prediction of LV functional recovery and remodeling [20,21]. However, there is a paucity of data examining the longitudinal changes in GLS over time in post-AMI patients, particularly in relation to HRV parameters.

The primary aim of our article is to evaluate HRV, LVEF, and GLS in patients following an AMI who were managed conservatively without primary percutaneous coronary intervention (PCI) or thrombolysis. Specifically, we aim to investigate the longitudinal changes in HRV and GLS post-AMI and analyze how these parameters correlate with myocardial performance and ANS function.

We hypothesized that decreased HRV and impaired GLS will be associated with worse cardiac outcomes, including arrhythmias and mortality. Additionally, we sought to identify early indicators of higher risk for adverse events by comparing HRV and cardiac performance parameters between deceased and surviving patients. By addressing these objectives, this study aims to enhance the understanding of post-MI recovery, improve prognostic assessments, and potentially guide the management and follow-up care of patients who have experienced an MI.

## 2. Materials and Methods

### 2.1. Study Population

The study, conducted prospectively, included patients diagnosed with MI, who were managed conservatively. The diagnosis of MI was established based on the criteria outlined in the fourth universal definition of MI [22], covering both ST-elevation myocardial infarction (STEMI) and non-ST-elevation myocardial infarction (NSTEMI). These patients were managed conservatively in accordance with the European Society of Cardiology guidelines [23,24,25]. All patients in the study were treated conservatively either due to late presentation—defined as beyond the recommended time window for reperfusion therapy (primary PCI or thrombolysis for STEMI within 12 h, or early invasive intervention for NSTEMI within 24–72 h)—or because they were not eligible for early invasive strategies due to factors like hemodynamic stability or patient preference.

Patients were admitted to the Coronary Intensive Care Unit and cardiology department between January 2021 and December 2023. Each patient underwent a thorough clinical, biological, and paraclinical assessment, which included the evaluation of HRV parameters and echocardiographic measurements. Data were collected on demographic characteristics, medical history, and biological workup.

For the study group, a baseline evaluation was performed within the first week of hospital admission and a follow-up evaluation was conducted at one month and six months post-MI. During the one-year follow-up there were patients that died of SCD. In our study, we categorized the deaths as SCDs after thoroughly reviewing each patient’s clinical history, symptoms, and available medical data to ensure that no other immediate noncardiac conditions, acute decompensated heart failure, or recurrent ischemic events could explain the death. For further comparative analysis, participants were also divided into two groups: Survivors and Deceased. The Deceased group comprised patients that died within 1 year from the AMI and Survivors were patients that survived one year after the acute event. The inclusion criteria for the study were adult patients with confirmed AMI based on clinical presentation, ECG changes, and modified cardiac biomarkers who did not receive thrombolysis, percutaneous coronary intervention (PCI), inotrope, or vasopressor substances as part of the treatment. Patients were excluded from the study if they met any of the following criteria: underwent PCI or systemic thrombolysis, had cardiac implantable electronic devices or required a temporary or permanent pacemaker, chronic kidney disease requiring dialysis, severe chronic obstructive pulmonary disease (COPD) with oxygen dependency, acute vascular cerebral events, including ischemic or hemorrhagic stroke, severe comorbid conditions limiting life expectancy to less than six months, or an inability to provide informed consent.

### 2.2. Heart Rate Variability Analysis and Cardiac Performance Evaluation

Each patient underwent 24 h electrocardiographic (ECG) monitoring using a Holter monitor. Holter ECG monitoring was conducted at baseline within the first week of hospitalization. Subsequent ECG recordings at one month and six months were performed during a one-day hospital stay. The ECG recordings were examined to evaluate HRV, detect arrhythmias, and identify other electrical abnormalities. The Holter data were transferred to a computer and analyzed using Labtech Cardiospy software. All recordings underwent a thorough visual inspection, with a manual removal of artifacts to ensure that at least 20 h of valid data were retained.

HRV parameters were obtained using Labtech Cardiospy v5.03 software. The selected HRV parameters followed the guidelines of the European Society of Cardiology and the North American Society of Pacemaker and Electrophysiology, utilizing a linear method analysis [26]. The conventional linear methods employed included time and frequency domain analyses. The time domain parameters analyzed were the standard deviation of RR intervals (SDNN), the root mean square of successive RR interval differences (RMSSD), and the percentage of adjacent NN intervals differing by more than 50 milliseconds (PNN50). Frequency domain parameters included low frequency (LF, 0.04 to 0.15 Hz) and high frequency (HF, 0.15 to 0.4 Hz). The LF and HF bands were generated using either the fast Fourier transform algorithm or autoregressive modeling.

Echocardiography was conducted using the ESAOTE MyLabX8 Platform (ESAOTE, Genova, Italy) ultrasound systems equipped with a 1–5 MHz transducer. All participants underwent conventional two-dimensional echocardiography. For assessing left ventricular ejection fraction (LVEF), the standard Simpson’s bi-plane method was used to determine LV volumes. Two-dimensional speckle-tracking echocardiography was conducted using the 2-chamber, 3-chamber, and 4-chamber apical views to assess myocardial strain. The endocardial border was traced automatically for the region of interest at the end systole and was further reviewed and adjusted manually if needed to ensure accurate speckle tracking. Global longitudinal strain (GLS) was calculated as the average peak strain from the three apical views, with the region of interest encompassing the entire left ventricle. If speckle tracking could not be performed from a specific view, the GLS was calculated by averaging the measurements from the other available views [27].

### 2.3. Statistical Analysis

Descriptive statistics were used to summarize the demographic and clinical characteristics of the study population, with data expressed as mean (standard deviation, SD) for normally distributed variables and as median (interquartile range, IQR) or as median (first interquartile Q1–third interquartile Q3) for non-normally distributed variables. Categorical data were expressed as percentages. For HRV and cardiac performance parameters description across different time points, the Shapiro–Wilk test was used to assess the normality of the data.

The primary statistical tests used in this analysis included the Kruskal–Wallis test, used to compare HRV metrics between the Deceased and Survivor groups at different time points. The chi-squared statistic and corresponding *p*-values were calculated to determine the significance of differences between the groups; the Friedman test was used for repeated measures to assess changes in HRV metrics and cardiac performance parameters over time; the Wilcoxon signed-rank test with Bonferroni correction were used for post hoc pairwise comparisons for multiple comparisons; and the chi-square test of independence was utilized to examine the associations between SDNN groups and various categorical variables. Statistical analysis was performed using RStudio Version 4.4.0 and Microsoft Excel 365 (2021), and significance was set at *p* < 0.05.

The cardiology clinic conducted all of the examinations regarding HRV and echocardiography parameters. All participants provided their informed consent per the principles outlined in the Declaration of Helsinki. The study protocol received approval from the ethical committee at our institution.

## 3. Results

### 3.1. Study Group Description

The study included 108 patients. During the one-year follow-up, 14 patients (12.96%) died of SCD. The mean age of the study population was 67.10 (8.46) years, which indicated a fairly homogenous age distribution. The study population demonstrated a high prevalence of traditional cardiovascular risk factors such as hypertension, dyslipidemia, and diabetes. A significant proportion of the patients (62.96%) had hypertension, dyslipidemia was present in 73.14% of the patients, and 43.51% of the patients had diabetes. The sex distribution showed a higher proportion of males (62.03%) compared with females (37.94%). The male predominance and the significant proportion of patients with a personal (56.48%) or familial (51.85%) history of cardiovascular disease were consistent with the typical demographic profile of patients with MI. Additionally, a percentage of patients had other health conditions: 10.18% had a diagnosis of chronic obstructive pulmonary disease and 6.48% had a history of cerebrovascular accident. A proportion of 28.70% of the patients had creatinine levels greater than 1.5 mg%, indicating a certain degree of impaired kidney function. The high prevalence of smoking (44.44%) and obesity (41.66%) further highlighted the presence of modifiable risk factors in this population. A total of 37.96% of patients presented nonfatal, nonsustained arrhythmias at some point during the study. At discharge, the majority of patients were prescribed medications according to established guidelines for post-MI care [23,24,25]. Beta-blockers were administered to 87.03% of patients, underscoring their essential role in reducing mortality and enhancing long-term outcomes [25]. Angiotensin-converting enzyme inhibitors (ACEi) or angiotensin receptor blockers (ARB) were given to 63.88% of patients, angiotensin receptor–neprilysin inhibitors (ARNi) were prescribed to 11.11% of patients, and mineralocorticoid receptor antagonists (MRA) were used in 48.14% of cases, highlighting their importance in managing heart failure and reducing hospitalizations [25]. Sodium–glucose co-transporter type 2 inhibitors (SGLT2i) were provided to 23.14% of patients for their beneficial effects on cardiovascular health, reverse remodeling, and improved outcomes [28]. Amiodarone and ivabradine were less frequently used, with 13.88% and 10.18% of patients receiving these medications, respectively (Table 1).

### 3.2. HRV Analysis

**Figure 1:**
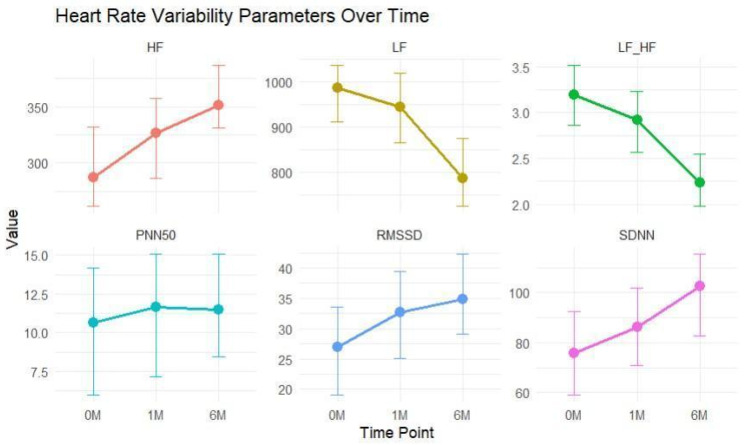
HRV parameters over time. SDNN: standard deviation of all RR intervals; RMSSD: root mean square of successive difference of RR; PNN50 (%): percent of successive RR differences > 50 ms; HF: high-frequency power; LF: low-frequency power; LF/HF: low-frequency/high-frequency ratio; 0M: baseline, 1M: 1 month; 6M: 6 months.

The medians of SDNN, RMSSD, PNN50, and HF increased over time, suggesting an improvement in HRV, which could indicate better autonomic regulation. Conversely, the LF and LF/HF ratio decreased over time, which could reflect a reduction in sympathetic activity or an improvement in autonomic balance. SDNN reflects overall HRV and is considered an indicator of autonomic balance, with higher values suggesting better cardiovascular health [8,9]. The significant increase in SDNN from baseline (75.3 (48.2–100)) to 1 month (87 (55.7–111)) (*p* < 0.001) indicated a marked improvement in autonomic function early after MI. Additionally, there was a significant increase from 1 month to 6 months (94.2 (67.6–118)) (*p* < 0.001), which suggested continuous recovery or improvement in autonomic regulation post-MI. RMSSD, which is a measure of short-term HRV and is associated with parasympathetic activity, showed significant increases at each time point, from baseline (27 (22–33)) to 1 month (30.5 (27.0–38.0)) (*p* < 0.001), from baseline to 6 months (35 (30–42)) (*p* < 0.001), and from 1 month to 6 months (*p* < 0.001). These results suggest a continuous progressive enhancement in parasympathetic activity. While there was a slight increase from baseline (10.6 (8.98–12.7)) to 1 month (11.1 (10.0–12.8)) (*p* = 0.047) for the PNN50 parameter, the more substantial increase from 1 month to 6 months (12.1 (9.7–16.8)) (*p* < 0.001) indicated an improvement in parasympathetic activity later following MI. Significant increases in HF power were observed from baseline (292 (266–321)) to 1 month (321 (293–348)) (*p* < 0.001), from baseline to 6 months (359 (320–384)) (*p* < 0.001), and from 1 month to 6 months (*p* < 0.001). These findings further supported the enhancement of parasympathetic activity over time. LF power is influenced by both sympathetic and parasympathetic activity but is often considered a marker of sympathetic activity. The LF power showed no significant change from baseline (959 (866–1043)) to 1 month (932 (840–1024)) (*p* = 0.540), which suggested a minimal immediate alteration in sympathetic activity post-MI. However, a significant decrease from baseline to 6 months (806 (688–916)) (*p* < 0.001) indicated a reduction in sympathetic activity in the later period after the acute event. The LF/HF ratio is an index of sympathovagal balance [9]. Significant reductions in the LF/HF ratio were observed at each time point (*p* < 0.001). These changes reflected a shift towards parasympathetic dominance or a more balanced ANS (Table 2 and Table 3, Figure 1).

### 3.3. Cardiac Performance Analysis

**Figure 2:**
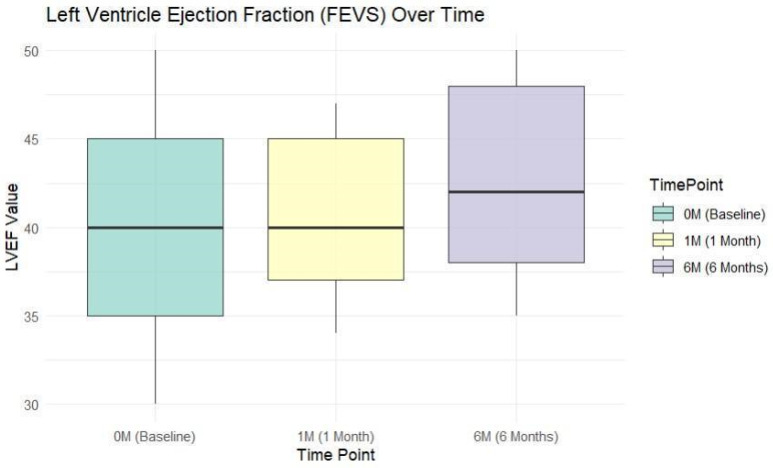
LVEF at different time points.

The median of the LVEF for the study group at baseline was 40% (17%), 40% (13%) at 1 month, and 42% (17%) at 6 months. The significant increase in LVEF from 1 month to 6 months post-infarction indicated a progressive improvement in left ventricular function over time and also a reduction in variability and more consistent improvements among patients. The changes in LVEF over the three time points indicated a significant improvement in LVEF over time (chi-squared: 53.59, DF: 2, *p* < 0.001) (Figure 2).

**Figure 3:**
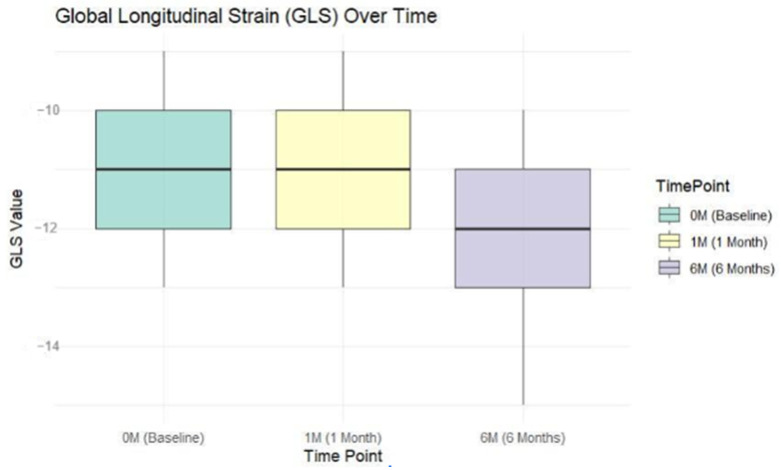
GLS at different time points.

The median of the GLS for the patients at baseline was −11% (5%), −11% (4%) at 1 month, and −13% (4%) at 6 months. The GLS values showed a significant improvement from baseline to 6 months post-infarction (chi-squared: 67.81, DF: 2, *p* < 0.001). The median GLS was stable from baseline to 1 month but improved significantly to −13% at 6 months (*p* < 0.001). The interquartile range (IQR) decreased from 5% at baseline to 4% at 1 month and remained stable at 4% at 6 months, indicating a reduction in variability and more consistent improvements among patients (Figure 3).

### 3.4. Associations between HRV, Cardiac Performance Analysis, and Different Risk Factors

This study also compared the associations between SDNN and the parameters for cardiac performance through LVEF and GLS. Patients were categorized into three groups based on their SDNN values at baseline: Group 1 (SDNN < 50 ms), Group 2 (SDNN between 50 and 100 ms), and Group 3 (SDNN > 100 ms).

**Figure 4:**
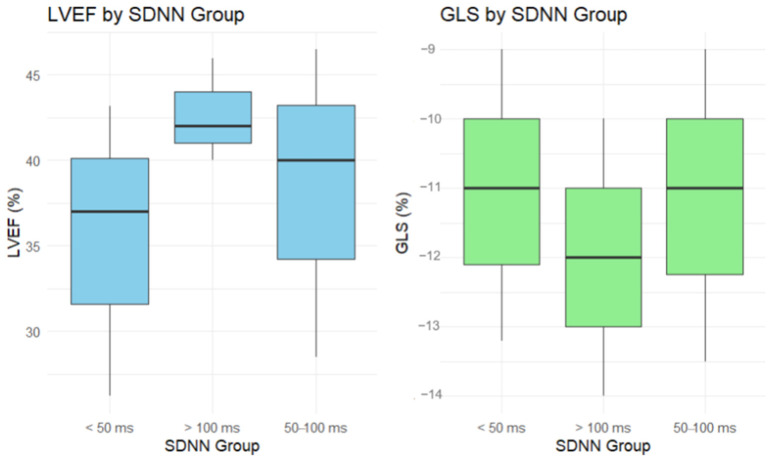
LVEF and GLS for different SDNN groups. SDNN: standard deviation of all RR intervals; LVEF: left ventricle ejection fraction; GLS: global longitudinal strain.

Regarding the SDNN groups there was a trend suggesting higher LVEF values with increasing SDNN, though the differences did not reach statistical significance (*p* = 0.071). Group 3, with SDNN > 100 ms, had the highest median LVEF (42%) and the lowest variability (IQR: 6%), indicating more consistent cardiac function compared with Groups 1 (37% (17%)) and 2 (40% (18%)). Median GLS values were slightly better in Group 3 (−12% (4%)) compared with Groups 1 and 2 at −11% (4.25%) and −11% (4.5%), respectively, but this difference was not statistically significant (*p* = 0.702) (Figure 4).

There was a significant association between the presence of arrhythmias and SDNN groups. The lower SDNN group (Group 1) had a higher observed count of arrhythmias when compared with the expected result, suggesting that patients with lower SDNN values are more likely to have arrhythmias, and indicating that SDNN may be a more relevant predictor for MI complications.

The association between hypertension, personal history of CVD, familial history of CVD, smoking status, dyslipidemia, obesity and diabetes, and SDNN groups, was not statistically significant. This indicated that the prevalence of these risk factors was similar across the different SDNN groups.

With further analysis of the SDNN groups and the presence of arrhythmias, significant differences were observed, with Group 1 (lower SDNN) showing a higher prevalence of arrhythmias when compared with Group 2 (moderate SDNN) (*p* = 0.00387) and Group 3 (high SDNN) (*p* < 0.001). No significant difference was observed, indicating that the prevalence of arrhythmias was similar between patients with moderate SDNN and high SDNN (*p* = 1). Lower SDNN values, especially < 50 ms, were associated with higher rates of arrhythmias, suggesting that very low HRV is linked to an increased risk of arrhythmias (Table 4).

The median SDNN values for Deceased patients were 48.55 ms at baseline, 64.2 ms at 1 month, and 70.8 ms at 6 months. For Survivors, the median values were higher, at 76.95 ms, 89.4 ms, and 99.05 ms, respectively. There were significant differences between the groups at all time points (*p* < 0.001), indicating that Survivors generally had higher HRV as measured by SDNN when compared with the Deceased patients. The median RMSSD values were 19.5 ms at baseline, 23 ms at 1 month, and 26.5 ms at 6 months for Deceased patients, while for Survivors the values were 29 ms, 33.5 ms, and 38 ms, respectively. Significant differences were observed between the groups at all time points (*p* < 0.001), suggesting better autonomic function in Survivors. For the PNN50, the median values for Deceased patients were 8.1% at baseline, 9.1% at 1 month, and 9.45% at 6 months. Survivors had higher median values of 11%, 11.6%, and 13.05%, respectively (*p* < 0.001), indicating more frequent normal variability in Survivors. HF medians were 211 at baseline, 238 at 1 month, and 216.5 at 6 months for Deceased patients. For Survivors, the medians were significantly higher: 301, 329.5, and 364.5, respectively, which showed significant differences (*p* < 0.001) and suggested greater parasympathetic activity in Survivors. Low-frequency power (LF) showed median values of 1109, 1112, and 1081 for Deceased patients, while Survivors had median values of 937.5, 903, and 788 (*p* < 0.001). The median LF/HF ratio was higher in Deceased patients (5.28, 4.64, 4.87) compared with Survivors (3.115, 2.755, 2.07) across all time points (*p* < 0.001), suggesting a higher sympathetic to parasympathetic balance in Deceased patients. The analysis of HRV metrics demonstrated significant differences between Deceased patients and Survivors across all measured parameters and time points. Survivors consistently showed higher HRV, indicating better autonomic function and balance (Table 5).

## 4. Discussion

The results in the present study indicate that HRV parameters generally improve over time following a MI, with significant changes detectable as early as one month post-event. This change may be reflective of better cardiovascular health and autonomic regulation. The improvement in HRV parameters, such as SDNN and RMSSD, over time is indicative of enhanced autonomic regulation, which is crucial for patient recovery and long-term cardiovascular health post-MI. Clinically, elevated HRV is associated with a lower risk of adverse outcomes, such as arrhythmias and mortality, making it a valuable marker for monitoring recovery. Similarly, the delayed but significant improvement in GLS from 1 month to 6 months post-AMI reflects important myocardial recovery and functional remodeling, which are critical for assessing the efficacy of therapeutic interventions and predicting patient prognosis. These clinical insights emphasize the importance of continued monitoring of HRV and GLS as part of post-AMI care to optimize patient outcomes and tailor individualized treatment strategies.

Overall, the significant changes observed in HRV parameters from baseline to 1 month and 6 months post-MI suggested a marked improvement in autonomic function. The increase in parasympathetic activity (as indicated by RMSSD, PNN50, and HF) and the decrease in sympathetic activity (as indicated by LF and LF/HF ratio) were consistent with improved autonomic balance. These findings underscored the utility of HRV as a valuable tool in monitoring and managing post-MI recovery, with potential implications for predicting long-term cardiovascular outcomes [29,30].

The LF/HF ratio’s significant reduction suggests a shift towards a more balanced ANS, potentially reducing the risk of future cardiac events. These findings support the use of HRV parameters as valuable indicators in the follow-up and management of post-MI patients. Our results are in accordance with the medical literature [6,31,32,33].

The conducted research showed that improved HRV metrics over time in MI patients were associated with better autonomic regulation and recovery post-MI. Lower HRV values were linked to higher arrhythmia rates and mortality. Goldenberg’s study [34] demonstrated that lower HRV is significantly associated with increased cardiovascular risk and myocardial ischemia in individuals without known coronary artery disease. Each unit reduction in HRV increased the likelihood of ischemia, while elevated HRV had a protective effect.

This work reported significant increases in SDNN, RMSSD, PNN50, and HF power from baseline to 6 months, indicating progressive enhancement in parasympathetic activity and reduced sympathetic activity. Lower SDNN values were significantly associated with a higher prevalence of arrhythmias, as compared to Goldenberg et al. [34], which showed that lower HRV was associated with an increased risk of cardiovascular events and also that an increase in the SDNN parameter resulted in a reduction in fatal or nonfatal cardiovascular events. Furthermore, this research found that survivors had higher HRV metrics compared with deceased patients at all time points, indicating better autonomic function and balance. Deceased patients had a higher LF power and LF/HF ratio, reflecting higher sympathetic activity. Both our study and Goldenberg’s study [34] support the use of HRV as a noninvasive marker for identifying individuals at higher risk of cardiovascular events. Lower HRV values were strongly linked to adverse outcomes, such as higher arrhythmia rates and mortality.

The present study showed significant differences in SDNN values between deceased patients and survivors. Survivors had consistently higher SDNN values, indicating better autonomic function. Lower SDNN was linked to higher mortality. The present data are in accordance with Buccelletti et al.’s study [35] that demonstrated that post-MI patients with SDNN < 70 ms had almost four times the risk of dying in the next three years compared with those with SDNN > 70 ms. The current investigation reported that survivors had higher median values for SDNN, RMSSD, PNN50, and HF power, indicating better parasympathetic activity and autonomic balance. Deceased patients had a higher LF power and LF/HF ratio, reflecting higher sympathetic activity. Buccelletti et al. [35] reinforced the importance of HRV as a marker for autonomic function, with low SDNN associated with worse outcomes. Buccelletti et al. [35] concluded that low SDNN is strongly associated with higher mortality post-MI. This is in accordance with our study that emphasizes the association of HRV with arrhythmias, cardiac performance, and survival, while Buccelletti et al.’s systematic review [35] provides robust evidence from multiple studies linking low SDNN to significantly higher mortality.

Huikuri et al.’s review [9] underlines the critical role of HRV in predicting adverse outcomes in post-MI patients. The present research focuses on the association of HRV with cardiac performance and arrhythmias, while Huikuri et al. showed HRV’s role in predicting SCD and arrhythmic events and reinforced the importance of HRV as a noninvasive risk marker for SCD, particularly when measured later, post-MI, emphasizing the timing of HRV measurement [9,36]. Our analysis showed significant differences in SDNN between deceased and surviving patients, with survivors having higher SDNN values, indicating better autonomic function and lower mortality risk.

There was a trend suggesting that there are higher left ventricular ejection fraction (LVEF) values with increasing SDNN, although these differences did not reach statistical significance. Patients with SDNN > 100 ms had the highest median LVEF. Studies showed that a significant association exists between lower SDNN and a higher prevalence of arrhythmias. Patients with SDNN < 50 ms showed a higher incidence of arrhythmias, indicating that very low HRV is linked to increased arrhythmic risk. In comparison, Chattipakorn et al. [37] showed that reduced HRV (SDNN < 70 ms) is a potent predictor of cardiovascular mortality in post-MI patients. HRV metrics have a strong predictive value for cardiac mortality and arrhythmic events. The prognostic significance of HRV depends on the timing of measurement. HRV measured early (within 2 weeks) and late (up to 1 year) post-MI has significant predictive value [37,38,39].

The investigation demonstrated significant improvements in LVEF over time in patients with MI. The increase in median LVEF from baseline to 6 months post-infarction indicated enhanced cardiac function and suggested a positive recovery trajectory for these patients. These improvements in LVEF were indicative of better overall cardiac health and improved prognosis for patients post-MI [15,16].

From baseline to 1 month, the GLS values remained relatively stable, suggesting that immediate post-infarction interventions could not have had a measurable impact on GLS within the first month. From 1 month to 6 months, there was a significant improvement in GLS. This suggested a delayed but notable improvement in myocardial function as the heart continued to recover post-infarction. This aligned with the existing medical literature, which depicted LV global functional recovery and remodeling following MI [15,20,21].

An unexpected finding in this analysis was the lack of statistically significant differences in GLS across different SDNN groups. Despite a trend suggesting that higher SDNN values might be associated with better GLS, this did not reach statistical significance. Similarly, while there was a trend towards higher LVEF with increasing SDNN, this difference also failed to achieve statistical significance. These results suggest that while SDNN may have an association with cardiac function parameters, such as LVEF and GLS, the relationships are not as strong as anticipated. This could suggest that other factors, beyond autonomic regulation as indicated by SDNN, could be influencing cardiac performance. This unexpected outcome highlighted the complexity of the interactions between HRV and cardiac performance metrics and underscored the need for larger studies to further elucidate these relationships and confirm our findings.

There were several limitations to this study. Extended ECG recordings are prone to artifacts and interference. Unlike short-term recordings, extended monitoring occurs in less controlled environments, which can pose challenges in maintaining signal quality. Variability in HRV measurements can occur due to factors like medication or inconsistent patient adherence to monitoring protocols. Similarly, GLS measurements can be influenced by the quality of echocardiographic imaging and might have inter-operator variability. Despite these limitations, extended ECG recordings offered the advantage of recording parameter values and more comprehensive data on autonomic function over time. Additionally, although variability in HRV and GLS could occur due to a series of variables, these factors also reflected real-world clinical settings, enhancing the applicability and relevance of the findings. Furthermore, the relatively small sample size may have limited the generalizability of our findings, as it reduced the statistical power and the ability to detect smaller effects or trends. The classification of SCD was based on clinical circumstances, which can be difficult to define precisely without continuous ECG monitoring or ventricular electrograms from implantable cardioverter-defibrillators at the time of death. While we rigorously reviewed clinical histories and symptoms to exclude noncardiac causes, acute decompensated heart failure, or recurrent ischemic events, the absence of direct arrhythmic evidence limited the certainty of our SCD definition. We acknowledge this limitation and emphasize that our categorization was based on available clinical data and the exclusion of other immediate causes. Future research can explore and address these gaps.

## 5. Conclusions

Our study highlights the significant prognostic value of HRV parameters in post-MI patients. The associations between HRV and cardiac performance parameters, such as LVEF and GLS, suggest that better autonomic regulation is linked to improved cardiac function. The significant changes in HRV parameters from baseline through one month and six months post-MI provide a robust framework for assessing patient recovery and predicting adverse outcomes. Clinically, an SDNN value below 50 ms has been associated with a higher risk of arrhythmias and mortality, while values above 100 ms are indicative of better autonomic function and a lower risk of adverse outcomes. Similarly, RMSSD and PNN50 showed progressive increases, with values at 6 months indicating a sustained enhancement in parasympathetic activity. Monitoring GLS over time is crucial as it offers a dynamic view of recovery, enabling clinicians to adjust treatments based on the patient’s evolving cardiac function. Clinicians should consider incorporating regular HRV and GLS assessments into follow-up care to monitor the recovery trajectory of post-MI patients. In conclusion, HRV parameters and cardiac performance analysis through LVEF, and especially using LV GLS, present a valuable tool in patients post-acute MI, and allow for the prediction of LV and autonomic function recovery and remodeling.

## Figures and Tables

**Table 1:** Characteristics of study population.

Characteristics	Values: % (*n* = 108)
Age (years) *	67.10 (8.46) *
Sex	M: 62.03% F: 37.94%
Hypertension	62.96%
Familial history of CVD	51.85%
Personal history of CVD	56.48%
Smokers	44.44%
Dyslipidemia	73.14%
Obesity	41.66%
Diabetes	43.51%
COPD	10.18%
History of CVA	6.48%
Creatinine > 1.5 mg%	28.70%
Arrhythmias	37.96%
Medication at discharge	
Beta-blockers	87.03%
Amiodarone	13.88%
Ivabradine	10.18%
ACEi/ARB	63.88%
ARNi	11.11%
SGLT2i	23.14%
MRA	48.14%

* Mean (SD); SD: standard deviation; CVD: cardiovascular disease; COPD: chronic obstructive pulmonary disease; CVA: cerebrovascular accident; ACEi/ARB: angiotensin-converting enzyme inhibitors/angiotensin receptor blockers; ARNi: angiotensin receptor–neprilysin inhibitor; SGLT2i: sodium–glucose cotransporter 2 inhibitors; MRA: aldosterone receptor antagonists.

**Table 2:** HRV parameters at different time points.

Parameter	Baseline Median (Q1–Q3)	1 Month Median (Q1–Q3)	6 Months Median (Q1–Q3)	Statistic *	*p*-Value (*p*)
SDNN	75.3 (48.2–100)	87 (55.7–111)	94.2 (67.6–118)	157.53	*p* < 0.001
RMSSD	27 (22–33)	30.5 (27.0–38.0)	35 (30–42)	190.75	
PNN50	10.6 (8.98–12.7)	11.1 (10.0–12.8)	12.1 (9.7–16.8)	33.47	
HF	292 (266–321)	321 (293–348)	359 (320–384)	131.98	
LF	959 (866–1043)	932 (840–1024)	806 (688–916)	54.54	
LF/HF	3.21 (2.89–3.56)	2.86 (2.54–3.17)	2.17 (1.87–2.63)	190.90	

SDNN: standard deviation of all RR intervals; RMSSD: root mean square of successive difference of RR; PNN50 (%): percent of successive RR differences > 50 ms; HF: high frequency power; LF: low frequency power; LF/HF: low-frequency/high-frequency ratio; Q1: first quartile; Q3: third quartile; * Friedman test.

**Table 3:** HRV parameters across different time points and the differences between time points.

Parameter (P)	Comparison of P Time Point	Compared with P Time Point	Statistic *	*p*-Value (*p*)
SDNN	Baseline Baseline 1 Month	1 Month 6 Months 6 Months	174 144 732	<0.001
RMSSD	Baseline Baseline 1 Month	1 Month 6 Months 6 Months	275 19.5 150	<0.001
PNN50	Baseline Baseline 1 Month	1 Month 6 Months 6 Months	2070 928 1266	0.016 <0.001 <0.001
HF	Baseline Baseline 1 Month	1 Month 6 Months 6 Months	167 240 1188	<0.001
LF	Baseline Baseline 1 Month	1 Month 6 Months 6 Months	2338 960 658	0.540 <0.001 <0.001
LF/HF	Baseline Baseline 1 Month	1 Month 6 Months 6 Months	5886 5843 5622	<0.001

SDNN: standard deviation of all RR intervals; RMSSD: root mean square of successive difference of RR; PNN50 (%): percent of successive RR differences > 50 ms; HF: high frequency power; LF: low frequency power; LF/HF: low-frequency/high-frequency ratio; * post hoc pairwise comparisons andusing the Wilcoxon signed-rank test with Bonferroni correction.

**Table 4:** Association of risk factors with SDNN groups.

Variable	SDNN Group (OC, EC)			Chi-Square Statistic *	*p*-Value (*p*)
	Group 1	Group 2	Group 3		
Arrhythmias	24, 13.67	12, 16.32	5, 11.01	6.52	0.0384
Hypertension	23, 22.67	25, 27.07	20, 18.26	0.89	0.6407
Personal history of CVD	18, 20.33	26, 24.29	17, 16.38	0.94	0.6229
Familial history of CVD	15, 18.67	24, 22.3	17, 15.04	2.29	0.3169
Smokers	15, 16	20, 19.11	13, 12.89	0.18	0.91
Dyslipidemia	25, 26.33	31, 31.45	23, 21.21	0.83	0.6582
Obesity	10, 15	18, 17.92	17, 12.08	6.28	0.052
Diabetes	15, 15.67	20, 18.71	12,12.62	0.26	0.8777

SDNN: standard deviation of all RR intervals; OC: observed counts; EC: expected counts; Group 1 (SDNN < 50 ms); Group 2 (SDNN between 50 and 100 ms); Group 3 (SDNN > 100 ms); * chi-square test of independence.

**Table 5:** Comparison of HRV metrics between Deceased and Survivors.

Parameter	Group	Baseline Median (IQR)	1 Month Median (IQR)	6 Months Median (IQR)	Kruskal–Wallis Chi-Squared	*p*-Value (*p*)
SDNN	Deceased	48.55 (24.15)	64.2 (44.07)	70.8 (29.5)	6.86	0.00878
	Survivors	76.95 (53.82)	89.4 (54.22)	99.05 (50.87)	6.53	0.01057
					11.57	<0.001
RMSSD	Deceased	19.5 (4.75)	23 (8.5)	26.5 (5.75)	17.55	<0.001
	Survivors	29 (10)	33.5 (11)	38 (10)	26.27	<0.001
					32.43	<0.001
PNN50	Deceased	8.1 (2.4)	9.1 (2.45)	9.45 (2.1)	22.36	<0.001
	Survivors	11 (3.375)	11.6 (2.65)	13.05 (6.325)	19.68	<0.001
					20.26	<0.001
HF	Deceased	211 (53	238 (51)	216.5 (40.5)	31.44	<0.001
	Survivors	301 (49.5)	329.5 (50.25)	364.5 (46.75)	33.42	<0.001
					36.22	<0.001
LF	Deceased	1109 (153.75)	1112 (147)	1081 (89.25)	23.99	<0.001
	Survivors	937.5 (172.75)	903 (169)	788 (170.75)	32.10	<0.001
					36.22	<0.001
LF/HF	Deceased	5.28 (0.68)	4.64 (0.46)	4.87 (0.57)	36.11	<0.001
	Survivors	3.115 (0.61)	2.75 (0.52)	2.07 (0.67)	36.17	<0.001
					36.22	<0.001

SDNN: standard deviation of all RR intervals; RMSSD: root mean square of successive difference of RR; PNN50 (%): percent of successive RR differences > 50 ms; HF: high-frequency power; LF: low-frequency power; LF/HF: low-frequency/high-frequency ratio; IQR: interquartile range.

## Data Availability

Data are contained within the article.
